# An Ultrastable Porous Polyhedral Oligomeric Silsesquioxane/Tetraphenylthiophene Hybrid as a High-Performance Electrode for Supercapacitors

**DOI:** 10.3390/molecules27196238

**Published:** 2022-09-22

**Authors:** Mohsin Ejaz, Mohamed Gamal Mohamed, Santosh U. Sharma, Jyh-Tsung Lee, Chih-Feng Huang, Tao Chen, Shiao-Wei Kuo

**Affiliations:** 1 Department of Materials and Optoelectronic Science, College of Semiconductor and Advanced Technology Research, National Sun Yat-sen University, Kaohsiung 80424, Taiwan; 2 Chemistry Department, Faculty of Science, Assiut University, Assiut 71515, Egypt; 3 Department of Chemistry, National Sun Yat-sen University, Kaohsiung 80424, Taiwan; 4 International PhD Program for Science, Department of Chemistry, National Sun Yat-sen University, Kaohsiung 80424, Taiwan; 5 Department of Medicinal and Applied Chemistry, Kaohsiung Medical University, Kaohsiung 807, Taiwan; 6 Department of Chemical Engineering, i-Center for Advanced Science and Technology (iCAST), National Chung Hsing University, 145 Xingda Road, South District, Taichung 40227, Taiwan; 7 Ningbo Institute of Material Technology and Engineering, Chinese Academy of Sciences, Ningbo 315201, China

**Keywords:** octavinylsilsesquioxane, porous organic inorganic polymers, thermal stability, cyclic voltammetry, tetraphenylthiophene, supercapacitor

## Abstract

In this study, we synthesized three hybrid microporous polymers through Heck couplings of octavinylsilsesquioxane (OVS) with 2,5-bis(4-bromophenyl)-1,3,4-oxadiazole (OXD-Br_2_), tetrabromothiophene (Th-Br_4_), and 2,5-bis(4-bromophenyl)-3,4-diphenylthiophene (TPTh-Br_2_), obtaining the porous organic–inorganic polymers (POIPs) POSS-OXD, POSS-Th, and POSS-TPTh, respectively. Fourier transform infrared spectroscopy and solid state ^13^C and ^29^Si NMR spectroscopy confirmed their chemical structures. Thermogravimetric analysis revealed that, among these three systems, the POSS-Th POIP possessed the highest thermal stability (T_5_: 586 °C; T_10_: 785 °C; char yield: 90 wt%), presumably because of a strongly crosslinked network formed between its OVS and Th moieties. Furthermore, the specific capacity of the POSS-TPTh POIP (354 F g^−1^) at 0.5 A g^−1^ was higher than those of the POSS-Th (213 F g^−1^) and POSS-OXD (119 F g^−1^) POIPs. We attribute the superior electrochemical properties of the POSS-TPTh POIP to its high surface area and the presence of electron-rich phenyl groups within its structure.

## 1. Introduction

**Scheme 1:**
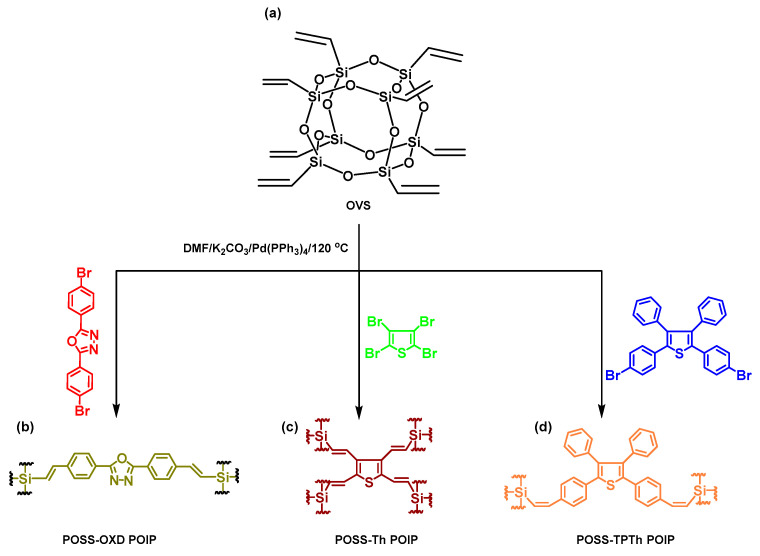
Synthesis of the (**b**) POSS-OXD, (**c**) POSS-Th, and (**d**) POSS-TPTh POIPs through Heck reactions of (**a**) OVS.

Porous organic polymers (POPs) are multidimensional materials featuring strong covalent bonds among their organic building blocks. They have recently gained popularity in the field of porous materials because of their low density, high porosity, and excellent thermal stability [1,2,3,4,5,6,7,8,9,10]. POPs have found applications in, for example, energy storage, lithium-ion batteries, gas separation, nanofiltration, drug carrying, optoelectronics, photocatalysis, and water treatment [11,12,13,14,15,16,17,18,19,20,21,22,23,24,25,26,27,28]. They have been synthesized using various techniques, including Friedel–Crafts, Yamamoto, Schiff base, Sonogashira, Yamamoto, Heck, and Suzuki reactions [29,30,31,32,33,34,35,36,37,38,39,40,41,42]. Furthermore, POPs comprise many subgroups of materials, including metal–organic frameworks, conjugated microporous polymers, covalent organic frameworks, hypercrosslinked polymers, polymers of intrinsic microporosity, and covalent triazine-based frameworks [20,21,22,23,24,25]. Hybrid porous materials incorporating polyhedral oligomeric silsesquioxane (POSS) units are interesting because of their outstanding mechanical and thermal characteristics and high surface areas [42,43,44,45,46,47]. POSS derivatives are nanosized compounds having diameters of 1–3 nm, with regulated porosity and defined structures [48,49,50,51,52,53,54,55,56]. Many POPs synthesized from POSS derivatives have exhibited remarkable thermal stabilities, fluorescence characteristics, and surface areas [56,57,58,59,60,61,62,63,64,65]. The boom in wearable and portable electronics has led to tremendous demand for cheap, eco-friendly, and high-performance energy-storage appliances [66,67,68,69,70,71,72]. The most crucial factors for a high-energy storage system are excellent energy density, safe operation, fabrication from accessible materials, high durability, and a prolonged life cycle. Although supercapacitors are energy storage systems that satisfy all these requirements [70,71,72], their energy densities have remained lower than those of batteries, thereby limiting their applications to date [73]. Incorporating redox-active groups into carbonaceous substances can enhance the performance of supercapacitors, potentially imparting them with functions that combine the mechanisms of pseudocapacitors and electrical double-layer capacitors (EDLCs) [74,75]. In this study, we synthesized three new hybrids porous POSS materials displaying excellent porosity and thermal stability, with potential for use in energy storage. Heck couplings of octavinylsilsesquioxane (OVS) with 2,5-bis(4-bromophenyl)-1,3,4-oxadiazole (OXD-Br_2_), tetrabromothiophene (Th-Br_4_), and 2,5-bis(4-bromophenyl)-3,4-diphenylthiophene (TPTh-Br_2_) in *N*,*N*-dimethylformamide (DMF) in the presence of a Pd catalyst and triethylamine (Et_3_N) as the base afforded the porous organic–inorganic polymers (POIPs) POSS-OXD, POSS-Th, and POSS-TPTh, respectively (Scheme 1). Herein, we describe their chemical structures, morphologies, pore sizes, thermal stabilities, electrochemical properties, and CO_2_ capture performance.

## 2. Synthesis Methods

### 2.1. Materials

Potassium carbonate (K_2_CO_3_), sodium bicarbonate (NaHCO_3_), and OVS were purchased from Alfa Aesar. 4-Bromobenzoyl bromide, hydrazine monohydrate (NH_2_NH_2_·H_2_O), methanol (MeOH), thiophene (Th), 1,4-dioxane (DO), tetrahydrofuran (THF), acetone, and DMF were procured from Acros. Benzeneboronic acid [PhB(OH)_2_], palladium tetrakis(triphenylphosphine) [Pd(PPh_3_)_4_], copper iodide (CuI), and Et_3_N were obtained from Sigma–Aldrich (Darmstadt, Germany).

### 2.2. N,N′-Bis(4-bromobenzoyl)hydrazine

A solution of 4-bromobenzoyl bromide (3.00 g, 11.3 mmol) and Et_3_N (3.75 mL, 27.0 mmol) in CHCl_3_ (50 mL) was cooled in an ice bath. NH_2_NH_2_·H_2_O (0.28 mL) was added and then the mixture was stirred for 10 h. The resultant white solid was washed sequentially with water and 10% NaHCO_3_ and then dried in an oven for 24 h (Yield: 80%, Scheme S1a). FTIR (Figure S1a): 3190 (NH), 1650 (C=O) cm^−1^. ^1^H NMR (Figure S2a): 10.67 (NH), 7.74–7.85 (ArH) ppm. ^13^C NMR (Figure S2c): 166.21 (C=O), 132.51–127.21 (aromatic carbon nuclei) ppm.

### 2.3. 2,5-Bis(4-bromophenyl)-1,3,4-oxadiazole (OXD-Br_2_)

A mixture of *N*,*N*′-bis(4-bromobenzoyl)hydrazine (2.50 g, 6.27 mmol) and POCl_3_ (74 mL) were heated at 80 °C for 24 h. The mixture was poured into cold water. The white needles were collected and washed with cold EtOH and water to afford OXD-Br_2_ (85%) (Scheme S1b). FTIR (Figure S1b): 3086 (C–H aromatic) cm^−1^. ^1^H NMR (Figure S2b): 8.07–7.85 (8H, ArH) ppm. ^13^C NMR (Figure S2d): 164.11 (C=N), 133.52–122.66 ppm.

### 2.4. 2,3,4,5-Tetrabromothiophene (Th-Br_4_)

Br_2_ (5.3 mL, 0.033 mmol) was added slowly to a solution of Th (2.0 g, 0.023 mmol) in CHCl_3_ (15 mL) at 0 °C and then the mixture was heated under reflux for 8 h. NaOH solution (2 M,100 mL) was added and then the mixture was heated under reflux for 8 h. The solid was filtered off and washed with water and EtOH to give Th-Br_4_ as a white powder (80%) (Scheme S2). FTIR (KBr, cm^−1^): 1636 (C=C). ^1^H NMR (500 MHz, CDCl_3_, δ, ppm, Figure S3): No peaks detected. ^13^C NMR (125 MHz, CDCl_3_, δ, ppm, Figure S4): 116.936, 110.284.

### 2.5. 2,3,4,5-Tetraphenylthiophene (TPTh)

A mixture of Th-Br_4_ (2.00 g, 1.40 mmol), Pd(PPh_3_)_4_ (0.300 g, 0.0500 mmol), 4-phenylboronic acid (3.70 g, 6.53 mmol), and K_2_CO_3_ (8.30 g, 16.6 mmol) was degassed three times under a flow of N_2_. DO (50 mL) and H_2_O (10 mL) were added and then the mixture was heated at 90 °C for 48 h. The solution was placed in ice-cold H_2_O and neutralized with HCl (2 mL). The solids were filtered off, washed with water and MeOH, and dried to produce a white powder (Scheme S3). FTIR (KBr, cm^−1^, Figure S5): 3063 (C–H aromatic), 1595 (C=C). ^1^H NMR (500 MHz, DMSO-*d*_6_, δ, ppm, Figure S6): 7.37–6.97 (16H, CH aromatic). ^13^C NMR (125 MHz, DMSO-*d*_6_, δ, ppm, Figure S7): 140.31–127.74 (Th and aromatic carbon nuclei).

### 2.6. 2,5-Bis(4-bromophenyl)-3,4-diphenylyhiophene (TPTh-Br_2_)

A solution of TPTh (5.00 g, 12.8 mmol) and Br_2_ (4.20 g) in CHCl_3_ (40 mL) was cooled at 0 °C (ice bath) for 4 h. After evaporation of the solvent, the residue was washed with EtOH to give a yellow solid (85%) (Scheme S4). FTIR (KBr, cm^−1^, Figure S8): 3047.38 (C–H aromatic). ^1^H NMR (500 MHz, DMSO-*d*_6_, δ, ppm, Figure S9): 7.37–6.81 (ArH). ^13^C NMR (125 MHz, DMSO-*d*_6_, δ, ppm, Figure S10): 140.87–121.97 (Th and aromatic carbon nuclei).

### 2.7. POSS-OXD, POSS-Th, and POSS-TPTh POIPs

A mixture of OVS (0.600 g, 0.950 mmol), OXD-Br_2_ (0.700 g, 1.84 mmol) [or Th-Br_4_ (0.750 g, 1.88 mmol) or TPTh-Br_2_ (0.86 g, 1.57 mmol)], anhydrous K_2_CO_3_ (2.09 g, 15.1 mmol), and Pd(PPh_3_)_4_ (0.0500 g, 0.0430 mmol) in DMF (50 mL) was heated under reflux for 48 h at 120 °C under N_2_. The resulting solid was collected, washed sequentially with MeOH, acetone, and THF, then dried in an oven to afford the POSS-OXD POIP (white powder, 0.45 g, 75%) [or the POSS-Th (white powder, 0.55 g, 92%) or POSS-TPTh (yellow powder, 0.51 g, 85%) POIPs].

## 3. Results

### 3.1. Synthesis and Character of POSS-OXD, POSS-Th and POSS-TPTh POIPs

Scheme 1 illustrates the syntheses of the three types of hybrid porous POSS frameworks: POSS-OXD, POSS-Th, and POSS-TPTh. We prepared OXD-Br_2_ through the reaction of *N*,*N*′-bis(4-bromobenzoyl)hydrazine with POCl_3_ at 80 °C (Scheme S1) and Th-Br_4_ through the reaction of Th with Br_2_ in CHCl_3_ at 0 °C (Scheme S2). We then obtained TPTh (Scheme S3) in high purity and yield through Suzuki coupling of Th-Br_4_ with PhB(OH)_2_ in toluene in the presence of Pd(PPh_3_)_4_ and K_2_CO_3_. Bromination of TPTh in CH_2_Cl_2_ in the presence of Br_2_ afforded TPTh-Br_2_ as a yellow solid (Scheme S4). Finally, we obtained the POSS-OXD, POSS-Th, and POSS-TPTh POIPs through Heck couplings of OXD-Br_2_, Th-Br_4_, and TPTh-Br_2_, respectively, with OVS in DMF in the presence of Pd(PPh_3_)_4_, Et_3_N, and K_2_CO_3_ at 120 °C for 48 h. These POSS-POIPs (Scheme 1) displayed poor solubility in all organic solvents, consistent with the successful Heck reactions of the OVS units and the formation of POSS materials with high degrees of crosslinking. 

**Figure 1:**
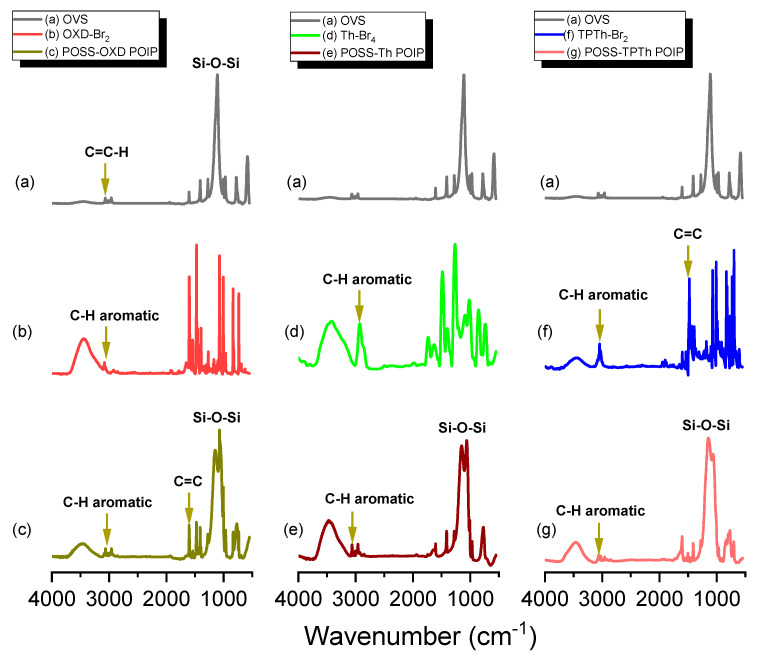
FTIR spectra of (**a**) OVS, (**b**) OXD-Br_2_, (**c**) POSS-OXD POIP, (**d**) Th-Br_4_, (**e**) POSS-Th POIP, (**f**) TPTh-Br_2_, and (**g**) POSS-TPTh POIP, recorded at 25 °C.

FTIR spectroscopy revealed the functional groups present in the synthesized porous POSS-POIP materials (Figure 1). The spectrum of OVS featured absorption bands at 3066, 1600, and 1106 cm^−1^, corresponding to the stretching of C=C–H, C=C, and Si–O–Si bonds, respectively (Figure 1a). The FTIR spectra of OXD-Br_2_, Th-Br_4_, TPTh-Br_2_, POSS-OXD POIP, POSS-Th POIP, and POSS-TPTh POIP revealed signals for aromatic C–H bonds in the range 3042–3082 cm^−1^ and for C=C stretching in the range 1600–1640 cm^−1^ (Figure 1b–g). In addition, the signals for the Si–O–Si units from all three porous POIPs were wider than that of OVS, consistent with the formation of crosslinked networks (Figure 1). In addition, the FTIR spectra of all the POIPs featured bands representing OH groups, resulting from their absorption of water. 

**Figure 2:**
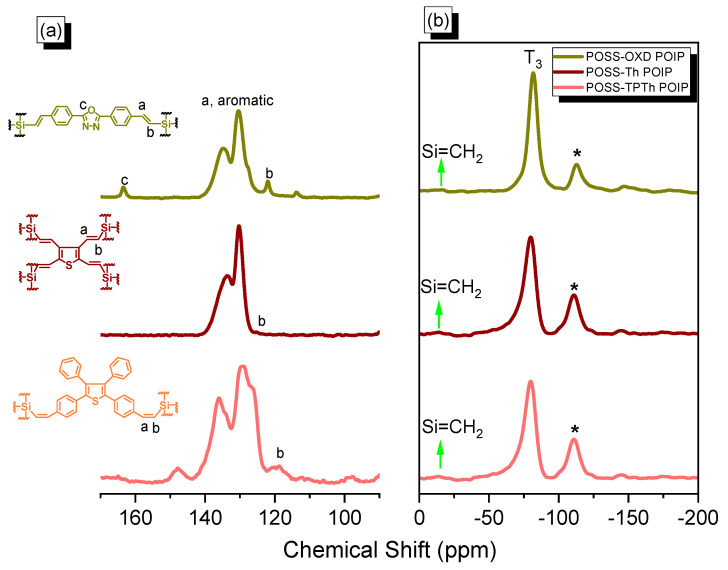
Solid state (**a**) ^13^C and (**b**) ^29^Si NMR spectra of the POSS-OXD, POSS-Th, and POSS-TPTh POIPs. * is the side band of solid state nuclear magnetic resonance spectroscopy (NMR).

The solid state ^13^C NMR spectra of the POIPs (Figure 2a) featured signals for their carbon nuclei in the ranges 121.7–134.85 ppm for the POSS-OXD POIP, 130.64–133.62 ppm for the POSS-Th POIP, and 118.42–136.14 ppm for the POSS-TPTh POIP, corresponding to the aromatic and SiCH=CH carbon nuclei of their framework structures. Solid state ^29^Si NMR spectroscopy confirmed the presence of POSS units in these POIPs frameworks (Figure 2b), with signals at −13.5 and −80 ppm assigned to Si=CH_2_ and T_3_ moieties, respectively. 

**Figure 3:**
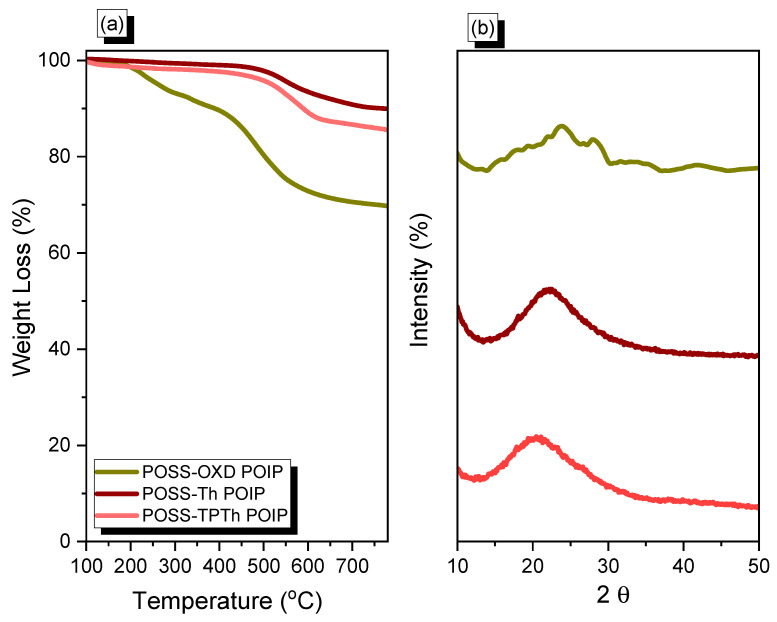
(**a**) TGA traces and (**b**) XRD patterns of the POSS-OXD, POSS-Th, and POSS-TPTh POIPs.

We used TGA to examine the thermal stabilities of the POSS-OXD, POSS-Th, and POSS-TPTh POIPs (Figure 3a). The thermal decomposition temperatures *T*_d5_ and *T*_d10_ and the char yields of the POSS-OXD POIP were 260 °C, 386 °C, and 69 wt%, respectively; for POSS-Th POIP, they were 586 °C, 786 °C, and 90 wt%, respectively; and for the POSS-TPTh POIP, they were 513 °C, 586 °C, and 86 wt%, respectively. For all three porous POIPs, these high values are consistent with the presence of highly crosslinked networks formed between the OVS and OXD, Th, and TPTh moieties. Notably, the thermal stability and char yield of the POSS-Th POIP were higher than those of the POSS-OXD and POSS-TPTh POIPs, as well as those of all other previously reported porous POSS systems [57,58], implying its higher crosslinking density. Table 1 summarizes the thermal properties of the POSS-OXD, POSS-Th, and POSS-TPTh POIPs. X-ray diffraction revealed that the POSS-OXD, POSS-Th, and POSS-TPTh POIPs were all amorphous materials with no long-range order in their characteristics (Figure 3b), consistent with data reported previously for porous hybrid POSS materials [57,58]. 

**Figure 4:**
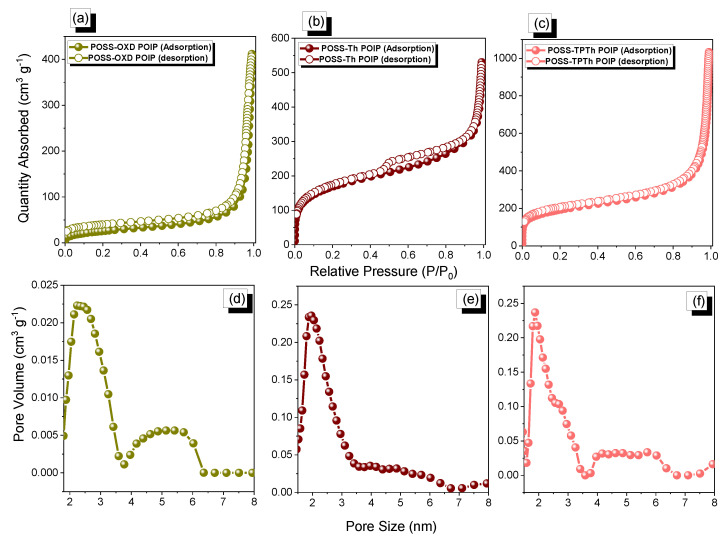
(**a**–**c**) N_2_ adsorption/desorption isotherms and (**d**–**f**) pore diameter patterns of the (**a**,**d**) POSS-OXD, (**b**,**e**) POSS-Th, and (**c**,**f**) POSS-TPTh POIPs.

We performed N_2_ adsorption/desorption measurements to obtain the porosity parameters of the POIPs (Figure 4a–c, Table 1). With reference to IUPAC classifications, the adsorption isotherm of the POSS-OXD POIP was of type III; that of POSS-Th POIP was of types II and IV; and that of POSS-TPTh POIP had type II characteristics. Furthermore, the N_2_ absorption uptake potentials for the POSS-Th and POSS-TPTh POIPs increased in the low- and high-pressure regions, confirming the existence of meso- and microspores in their structures; in contrast, POSS-OXD POIP had high N_2_ absorption uptake at high pressure, suggesting the presence of only macropores. The specific surface area of the POSS-TPTh POIP (682 m^2^ g^−1^) was higher than those of the POSS-Th (605 m^2^ g^−1^) and POSS-OXD (94 m^2^ g^−1^) POIPs. The pore diameters of the POSS-OXD, POSS-Th, and POSS-TPTh POIPs were 2.34, 1.96, and 1.88 nm, respectively (Figure 4d–f, Table 1); their total pore volumes were 0.02, 0.23, and 0.23 cm^3^ g^−1^, respectively. 

**Figure 5:**
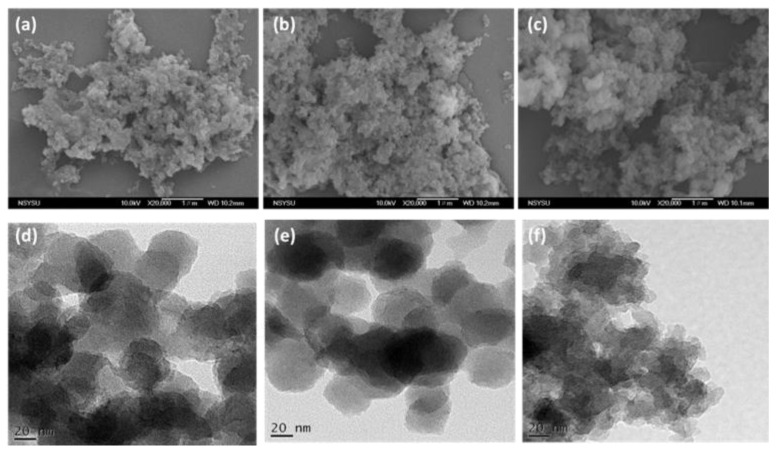
(**a**–**c**) SEM and (**d**–**f**) TEM images of the (**a**,**d**) POSS-OXD, (**b**,**e**) POSS-Th, and (**c**,**f**) POSS-TPTh POIPs.

Scanning electron microscopy (SEM) and transmission electron microscopy (TEM) revealed (Figure 5) that the morphologies of the POSS-OXD, POSS-Th, and POSS-TPTh POIPs were irregular clusters of tiny spherical particles. Furthermore, energy dispersive X-ray (EDX) spectroscopy of the SEM images (Figure S11) demonstrated the presence of C, N, O, and Si atoms in the POSS-OXD POIP and C, O, Si, and S elements in both the POSS-Th and POSS-TPTh POIPs. 

**Figure 6:**
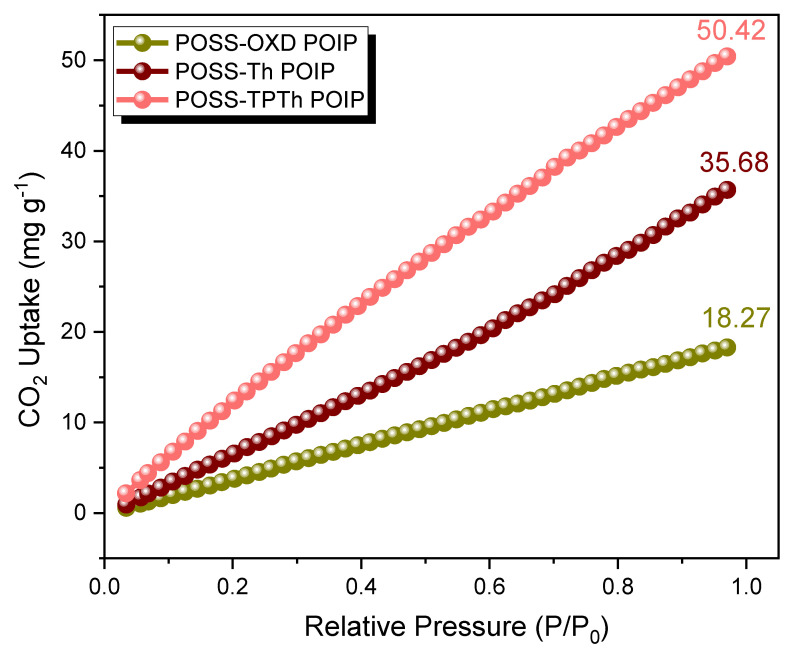
CO_2_ uptake capacities of the POSS-OXD, POSS-Th, and POSS-TPTh POIPs.

We tested these three porous POSS materials for their ability to capture CO_2_ at 298 °C (Figure 6). The POSS-OXD, POSS-Th, and POSS-TPTh POIPs had CO_2_ capture capacities of 18.27, 35.68, and 50.42 mg g^−1^, respectively. The relatively high CO_2_ capture capacity of the POSS-TPTh POIP was presumably due to its Brunauer–Emmett–Teller (BET) surface area being higher than those of the POSS-Th and POSS-OXD POIPs.

### 3.2. Electrochemical Performance of the POSS-OXD, POSS-Th, and POSS-TPTh POIPs

**Figure 7:**
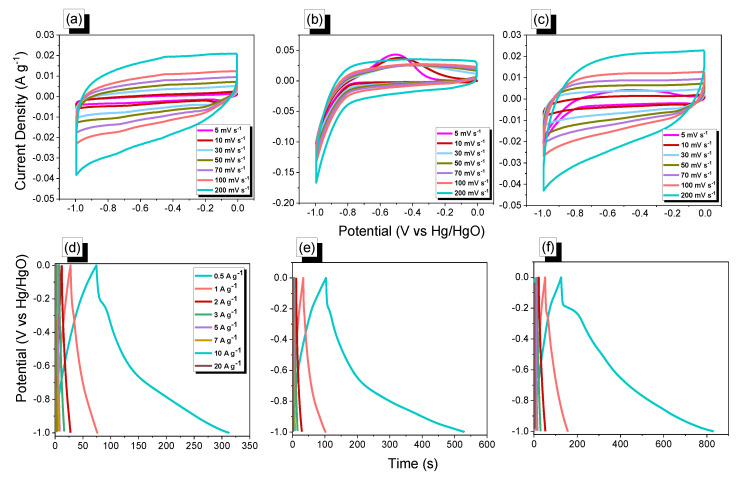
(**a**–**c**) CV and (**d**–**f**) GCD traces of the (**a**,**d**) POSS-OXD, (**b**,**e**) POSS-Th, and (**c**,**f**) POSS-TPTh POIPs.

We employed cyclic voltammetry (CV) and galvanostatic charge–discharge (GCD) measurements to examine the electrochemical behavior of our three POIPs in aqueous 1 M KOH, using a three-electrode configuration. We recorded the CV profiles of the POSS-OXD, POSS-Th, and POSS-TPTh POIPs at various scan rates between 1.00 to 0 V (Figure 7a–c). The rectangular CV profiles with slight humps for all three POSS systems indicate that their capacitive effects were caused by EDLC [76,77]. The humps suggest the involvement of pseudocapacitance responses emerging from the presence of the N and S heteroatoms and electron-rich phenyl groups in the OXD, Th, and TPTh structures, respectively, facilitating the passage of the electrolyte to the electrode area and for the electrons to keep moving from one electrode to another [78,79]. Upon increasing the scan rate, the current densities improved without affecting the shapes of the CV curves, confirming the high-rate capabilities, stabilities, and kinetic features of all three porous POSS systems. The electrochemical properties of the POSS-TPTh POIP were superior to those of the POSS-Th and POSS-OXD POIPs, presumably because of its electron-rich phenyl groups and S heteroatoms, high surface area, and blend of micro- and mesoporosity. All these factors would ensure higher mobility of the electrolyte to the surface of the electrode, resulting in accelerated mass transport and enhanced electrochemical performance. Figure 7d–f display the GCD behavior of the POSS-OXD, POSS-Th, and POSS-TPTh POIPs, respectively, measured at various current densities. In the discharge profiles, each porous POSS system provided a nearly triangular GCD curve with a small bend, indicating the combined impacts of pseudocapacitance and EDLC.

**Figure 8:**
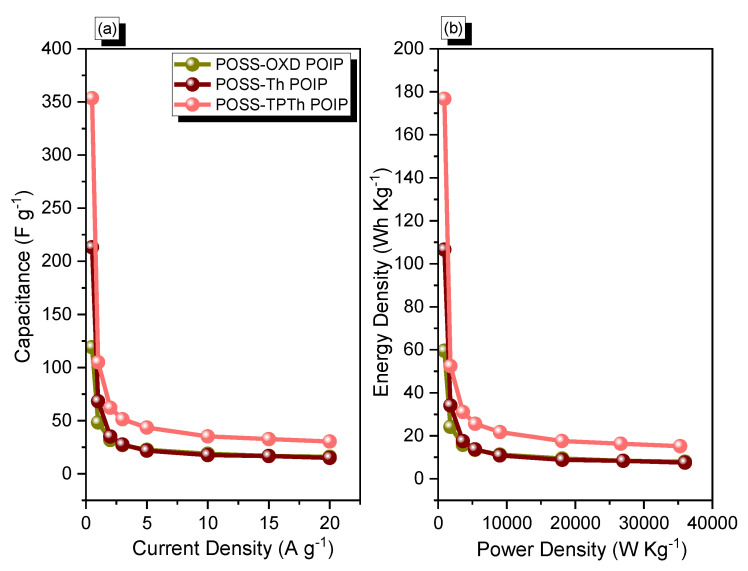
(**a**) Capacitance and (**b**) Ragone plots for the POSS-OXD, POSS-Th, and POSS-TPTh POIPs.

Furthermore, the charging profiles were smaller than the discharging curves throughout the GCD analyses, indicating that all the porous POIPs displayed excellent capacitance. The discharging profile of the POSS-TPTh POIP was more significant than those of the POSS-Th and POSS-OXD POIPs, indicating its relatively high specific capacitance. It is worth mentioning that the IR drop in the galvanostatic charge-discharge profile is related to the equivalent series resistance, charge-transfer resistance, and double-layer capacitance of the energy storage devices, such as supercapacitors. A supercapacitor has its own stored energy in the form of internal resistance which is lost during this process [80]. Figure 8a reveals that the specific capacitances of the POSS-OXD, POSS-Th, and POSS-TPTh POIPs were 119, 213, and 354 F g^−1^, respectively, at a current density of 0.5 A g^−1^. Furthermore, the specific capacitance gap remained large when measured at a higher current density of 20 A g^−1^, where the POSS-OXD, POSS-Th, and POSS-TPTh POIPs had specific capacities of 16, 15, and 30.4 F g^−1^, respectively. We suggest that the structure of the POSS-TPTh POIP was responsible for its relatively outstanding performance, with its electron-rich phenyl rings and S heteroatoms allowing the electrolytes to approach the electrode surface more rapidly than in the POSS-OXD and POSS-Th POIPs. The specific capacitances of all three porous POIPs decreased upon increasing the current density from 0.5 to 20 A g^−1^, presumably because of insufficient time for ion transportation at higher current densities. Moreover, the energy density of the POSS-TPTh POIP (176.75 W h kg^−1^) was also higher than those of the POSS-Th (106.59 W h kg^−1^) and POSS-OXD (59.57 W h kg^−1^) POIPs (Figure 8b). 

**Figure 9:**
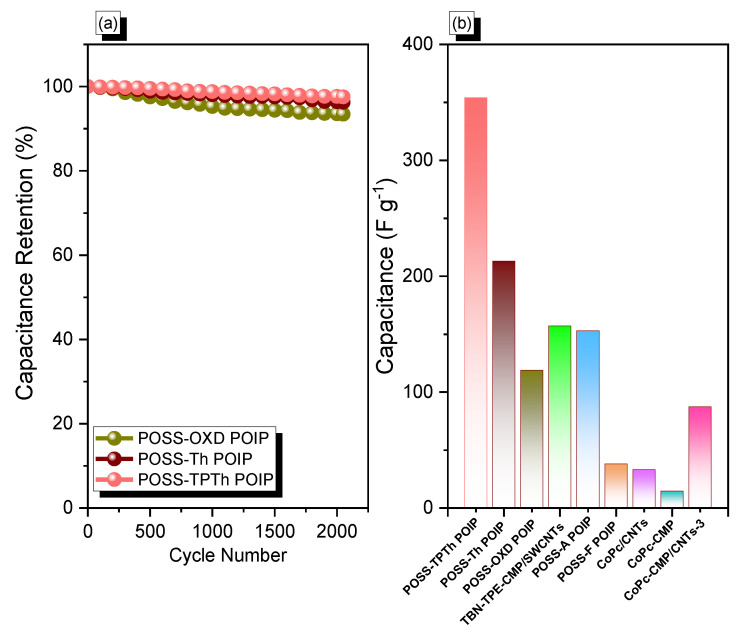
(**a**) Durability and (**b**) electrochemical performance of the POSS-OXD, POSS-Th, and POSS-TPTh POIPs, compared with those of previously reported porous materials.

Finally, we examined the stabilities of all three POIPs through GCD analyses over 2000 cycles at 10 A g^−1^ (Figure 9a and Figures S12–14). The POSS-OXD, POSS-Th, and POSS-TPTh POIPs displayed outstanding cycling stabilities, with retention of their capacitance of 93.41, 96.27, and 97.56%, respectively. In addition, the specific capacitances of both the POSS-Th and POSS-TPTh POIPs were excellent when compared with those of previously published porous frameworks and porous polymer composite materials (Figure 9b) [27,28,58]. 

Additionally, we investigated the electrochemical characteristics of all three POSS-POIP samples in two electrode systems for a symmetric supercapacitor through coin cells. The electrochemical characteristics were conducted between a potential range of 0 to −0.2 V at different scan rates, as shown in Figure S15. The CV profiles of all the POSS-POIPs electrodes (Figure S15a–c) were approximately rectangular with the presence of humps in the low potential window—the expected response of supercapacitors due to the existence of EDLC and pseudocapacitive capacitive characteristics. The pseudocapacitance response was caused by the introduction of heteroatoms (N, O, and S) in the backbone of the POSS-POIPs. The functionality of the electrodes seemed to be stable at high scan rates, indicating that the current density also increased, revealing improved stability and a symmetric capacity of all POSS-POIPs materials. Furthermore, the GCD profiles (Figure S15d–f) were nearly triangular with a bent shape due to the presence of heteroatoms in all the POSS-POIPs, demonstrating EDLC and pseudocapacitance effects. The specific capacitance was observed as 41.05, 27.63 and 7.11 F g^−1^ for POSS-TPTh POIP, POSS-Th POIP, and POSS-OXD POIP electrode samples, respectively, at 0.5 A g^−1^. Thus, these results revealed that POSS-TPTh POIP experienced superior electrochemical properties to POSS-Th POIP, and POSS-OXD POIP. The superior electrochemical properties of the POSS-TPTh POIP sample were attributed to its high surface area and electron-rich phenyl group in its structure. In order to understand the resistance behavior of these as-prepared electrodes as symmetric supercapacitors devices, we carried out the impedance measurement, as shown in Figure S16. The EIS plots reveal the ohmic resistance, which is also known as the series resistance, of these electrode materials to be 2.083, 3.799 and 4.491 Ω for POSS-TPTh POIP, POSS-OXD POIP and POSS-Th POIP, respectively. This confirms the superior performance of the POSS-TPTh POIP electrodes compared to the others by virtue of their smallest series resistance offering better conductivity. Apart from this, all the electrodes displayed a clear semicircle in the higher frequency region which corresponds to their charge-transfer resistance. As indicated in the plots, the charge-transfer values obtained from these electrodes were 4.479, 13.72 and 20.16 Ω, for POSS-TPTh POIP, POSS-OXD POIP and POSS-Th POIP, respectively. The electrode composed of POSS-TPTh POIP offers almost negligible charge-transfer resistance, with improved conductivity compared to the others. Thus, POSS-TPTh POIP is an outstanding electrode material for energy storage which has the potential to be used in real-life applications.

## 4. Conclusions

In conclusion, we used Heck reactions to synthesize the hybrid OVS-based POIPs POSS-OXD, POSS-Th, and POSS-TPTh. TGA revealed that the thermal stability of the POSS-Th POIP (T_10_: 785 °C; char yield: 90 wt%) was higher than those of the POSS-OXD and POSS TPTh POIPs. N_2_ isothermal profiles and electrochemical analyses indicated that POSS-TPTh POIP had the highest BET surface area (682 m^2^ g^−1^) and an excellent specific capacity (354 F g^−1^) when compared with those of the other two POIPs. The superior energy storage performance of the POSS-TPTh POIP was due to its high surface area and electron-rich phenyl groups, which were not present in the POSS-OXD and POSS-Th POIPs. In addition to this, we evaluated the electrochemical performance of a symmetric supercapacitor two-electrode device which exhibited great potential for energy-storage. These findings suggest that the POSS-TPTh and POSS-Th POIP materials, with their high surface areas, might find application in wastewater treatment and in Li and Li-S batteries.

## Figures and Tables

**Table 1:** TGA, BET, and capacitance data for the POIPs.

Sample	*T*_d10_ (°C)	Char Yield (wt.%)	Surface Area (m^2^ g^−1^)	Pore Volume (cm^3^ g^−1^)	Pore Size (nm)	Capacity at 0.5 A g^−1^ (F g^−1^)
**POSS-OXD POIP**	386	69	94	0.02	2.34	119
**POSS-Th POIP**	786	90	605	0.23	1.98	213
**POSS-TPTh POIP**	586	86	682	0.23	1.87	354

## Data Availability

The data presented in this study are available in the article and supplementary material.

## Supplementary Materials

The following are available online: https://www.mdpi.com/article/10.3390/molecules27196238/s1, Scheme S1. Syntheses of (a) *N*,*N*′-bis(4-bromobenzoyl)hydrazine and (b) OXD-Br_2_. Scheme S2. Synthesis of Th-Br_4_ through bromination reaction of thiophene. Scheme S3. Synthesis of TPTh from Th-Br_4_ through Suzuki coupling reaction. Scheme S4. Synthesis of TPTh-Br_2_ from Th-4Ph through bromination reaction. Figure S1. FTIR spectra of (a) *N*,*N*′-bis(4-bromobenzoyl)hydrazine and (b) OXD-Br_2_. Figure S2. (a,b) ^1^H and (c,d) ^13^C NMR spectra of (a,c) *N*,*N*′-bis(4-bromobenzoyl)hydrazine and (b,c) OXD-Br_2_. Figure S3. ^1^H NMR spectrum of Th-Br_4_. Figure S4. ^13^C NMR spectrum of Th-Br_4_. Figure S5. FTIR spectrum of TPTh. Figure S6. ^1^H NMR spectrum of TPTh. Figure S7. ^13^C NMR spectrum of TPTh. Figure S8. FTIR analysis of TPTh-Br_2_. Figure S9. ^1^H NMR analysis of TPTh-Br_2_. Figure S10. ^13^C NMR analysis of TPTh-Br_2_. Figure S11. SEM and elemental mapping (EDX) profile of the as-prepared (a) POSS-OXD POIP, (b) POSS-Th POIP and (c) POSS-TPTh POIP. Figure S12. Stability test of the as-prepared POSS-OXD POIP. Figure S13. Stability test of the as-prepared POSS-Th POIP. Figure S14. Stability test of the as-prepared POSS-TPTh POIP. Figure S15. Cyclic voltammetry curves (a–c) and (d–f) galvanostatic charge-discharge profiles of POSS-OXD, POSS-Th and POSS-TPTh POIPs. Figure S16. Nyquist plots of POSS-Th, POSS-TPTh, and POSS-OXD POIPs.

## Sample Availability

Samples of the compounds are not available from the authors.
