# Supplementary material for: An Ultrastable Porous Polyhedral Oligomeric Silsesquioxane/Tetraphenylthiophene Hybrid as a High-Performance Electrode for Supercapacitors
Source: Molecules. 2022 Sep 22;27(19):6238. doi: 10.3390/molecules27196238 (PMC9572779; doi:10.3390/molecules27196238)
Supplement: Supplementary file 1 [file molecules-27-06238-s001.zip › molecules-1909558-supplementary.pdf]

Supporting Information for

An Ultrastable Porous Polyhedral Oligomeric Silsesquioxane/Tetraphenylthiophene Hybrid as a High-Performance Electrode for Supercapacitors

Mohsin Ejaz [1], Mohamed Gamal Mohamed [1,2,*], Santosh U. Sharma [3,4], Jyh-Tsung Lee [3,4,7], Chih-Feng Huang [5], Tao Chen [6] and Shiao-Wei Kuo [1,7,*]

[1] Department of Materials and Optoelectronic Science and College of Semiconductor and Advanced Technology Research, National Sun Yat-Sen University, Kaohsiung 80424, Taiwan
[2] Chemistry Department, Faculty of Science, Assiut University, Assiut 71515, Egypt
[3] Department of Chemistry, National Sun Yat-Sen University, Kaohsiung 80424, Taiwan
[4] International PhD Program for Science, Department of Chemistry, National Sun Yat-Sen University, Kaohsiung 80424, Taiwan
[5] Department of Chemical Engineering, i-Center for Advanced Science and Technology (iCAST), National Chung Hsing University, 145 Xingda Road, South District, Taichung 40227, Taiwan
[6] Ningbo Institute of Material Technology and Engineering, Chinese Academy of Science, Ningbo, 315201, China
[7] Department of Medicinal and Applied Chemistry, Kaohsiung Medical University, Kaohsiung 807, Taiwan
* Correspondence: mgamal.eldin34@gmail.com (M.G.M.); kuosw@faculty.nsysu.edu.tw (S.-W.K.)

Characterization

FTIR spectra were collected using a Bruker Tensor 27 FTIR spectrophotometer at a resolution of 4 cm^{-1} and the KBr disk method. ^{13}C nuclear magnetic resonance (NMR) spectra were recorded using an INOVA 500 instrument, with DMSO as the solvent and tetramethylsilane (TMS) as the external standard; chemical shifts are reported in parts per million (ppm). The thermal stabilities of the samples under N_2 were measured using a TG Q-50 thermogravimetric analyzer; the cured sample (ca. 5 mg) was placed in a Pt cell and then heated at 20 °C min^{-1} from 100 to 800 °C under a N_2 flow of 60 mL min^{-1}. Wide-angle X-ray diffraction (WAXD) patterns were measured at the wiggler beamline BL17A1 of the National Synchrotron Radiation Research Center (NSRRC), Taiwan; a triangular bent Si

(111) single crystal was used to obtain a monochromated beam having a wavelength (λ) of 1.33 Å. The morphologies of the polymer samples were examined through field emission scanning electron microscopy (FE-SEM; JEOL JSM7610F) and transmission electron microscopy (TEM), using a JEOL-2100 microscope operated at an accelerating voltage of 200 kV. BET surface areas and porosimetry measurements of the samples (ca. 40–100 mg) were performed using a BEL Master™ instrument and BEL sim™ software (v. 3.0.0); N_2 adsorption and desorption isotherms were generated through incremental exposure to ultrahigh-purity N_2 (up to ca. 1 atm) in a liquid N_2 (77 K) bath; surface parameters were calculated using the BET adsorption models in the instrument's software. The pore sizes of the prepared samples were determined using nonlocal density functional theory (NLDFT).

Electrochemical Analysis

Working Electrode Cleaning: Prior to use, the glassy carbon electrode (GCE) was polished several times with 0.05-μm alumina powder, washed with EtOH after each polishing step, cleaned through sonication (5 min) in a water bath, washed with EtOH, and then dried in air.

Electrochemical Characterization: The electrochemical experiments were performed in a three-electrode cell using an Autolab potentiostat (PGSTAT204) and 1 M KOH as the aqueous electrolyte. The GCE was used as the working electrode (diameter: 5.61 mm; 0.2475 cm^2); a Pt wire was used as the counter electrode; Hg/HgO (RE-1B, BAS) was the reference electrode. All reported potentials refer to the Hg/HgO potential. A slurry was prepared by dispersing the POSS-POIPs samples (45 wt. %), carbon black (45 wt. %), and Nafion (10

wt. %) in a mixture of (EtOH/ H_2O) (200 μL: 800 μL) and then sonicating for 1 h. A portion of this slurry (10 μL) was pipetted onto the tip of the electrode, which was then dried in air for 30 min prior to use. The electrochemical performance was studied through CV at various sweep rates (5–200 mV s^{-1}) and through the GCD method in the potential range from 0 to –1.00 V (vs. Hg/HgO) at various current densities (0.5–20 A g^{-1}) in 1 M KOH as the aqueous electrolyte solution.

The specific capacitance was calculated from the GCD data using the equation:

$$C_s = (I\Delta t)/(m\Delta V) \quad \textbf{(S1)}$$

Where C_s (F g^{-1}) is the specific capacitance of the supercapacitor, I (A) is the discharge current, ΔV (V) is the potential window, Δt (s) is the discharge time, and m (g) is the mass of the NPC on the electrode. The energy density (E, W h kg^{-1}) and power density (P, W kg^{-1}) were calculated using the equations.

$$E = 1000C(\Delta V)^2/(2 \times 3600) \quad (\textbf{S2})$$

and

$$P = E/(t/3600) \quad (\textbf{S3})$$

We evaluated the electrochemical functionality of a symmetric supercapacitor using a CR2032 coin cell, which consists of an anode and cathode, a bottom and top cover, a metal spring, a separator, and an electrolyte. Our compounds served as both the cathode and the anode in order to construct a symmetric supercapacitor. The slurry was created by combining 2 mg of POSS-POIP, 2 mg of conductive carbon, 20 mL of nafion, 200 mL of

ethanol, and 400 mL of water. It was then sonicated for an hour and cast onto carbon paper. We used a Selemion AMV membrane with an electrolyte of 1.0 M aqueous KOH.

The specific capacitance was calculated in assembled supercapacitor from the GCD data using the following equations:

$C_s = 2(I\Delta t)/(m\Delta V)$ **(S4)**

where C_s (F g^{-1}) is the specific capacitance of the supercapacitor, I (A) is the discharge current, ΔV (V) is the potential window, Δt (s) is the discharge time, and m (g) is the mass of the CMP in single electrode.

The energy density (E, W h kg^{-1}) and power density (P, W kg^{-1}) were calculated using the equations:

$E_{cell} = 1000\ C_s\ (\Delta V)^2/(4 \quad 2 \quad 3600)$ **(S5)**

and

$P_{cell} = E_{cell}\ /(t/3600)$ **(S6)**

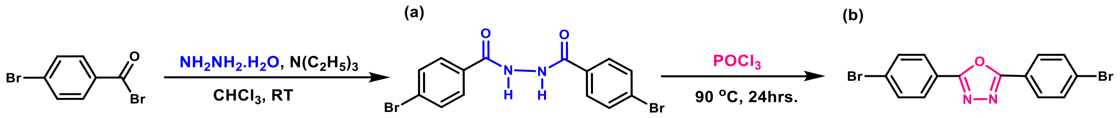

Scheme S1. Syntheses of (a) *N,N′*-bis(4-bromobenzoyl)hydrazine and (b) OXD-Br_2.

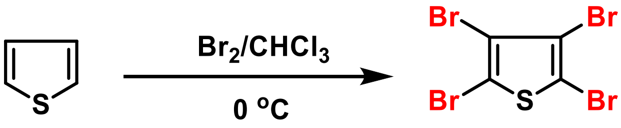

Scheme S2. Synthesis of Th-Br_4 through bromination reaction of Thiophene.

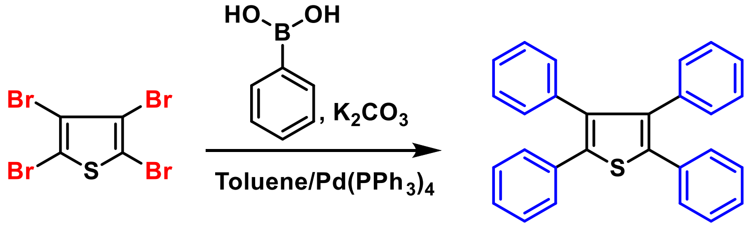

Scheme S3. Synthesis of TPTh from Th-Br_4 through Suzuki coupling reaction.

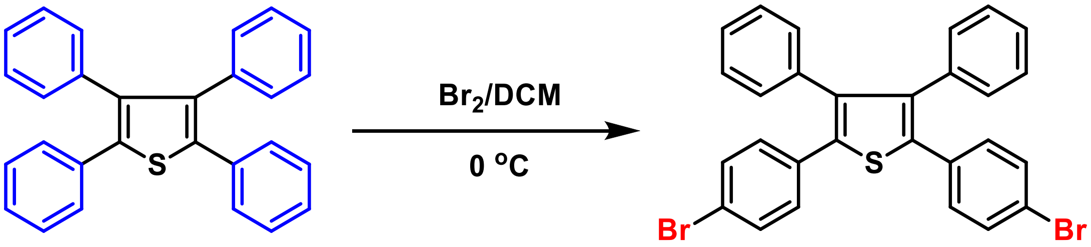

Scheme S4. Synthesis of TPTh-Br_2 from Th-4Ph through bromination reaction.

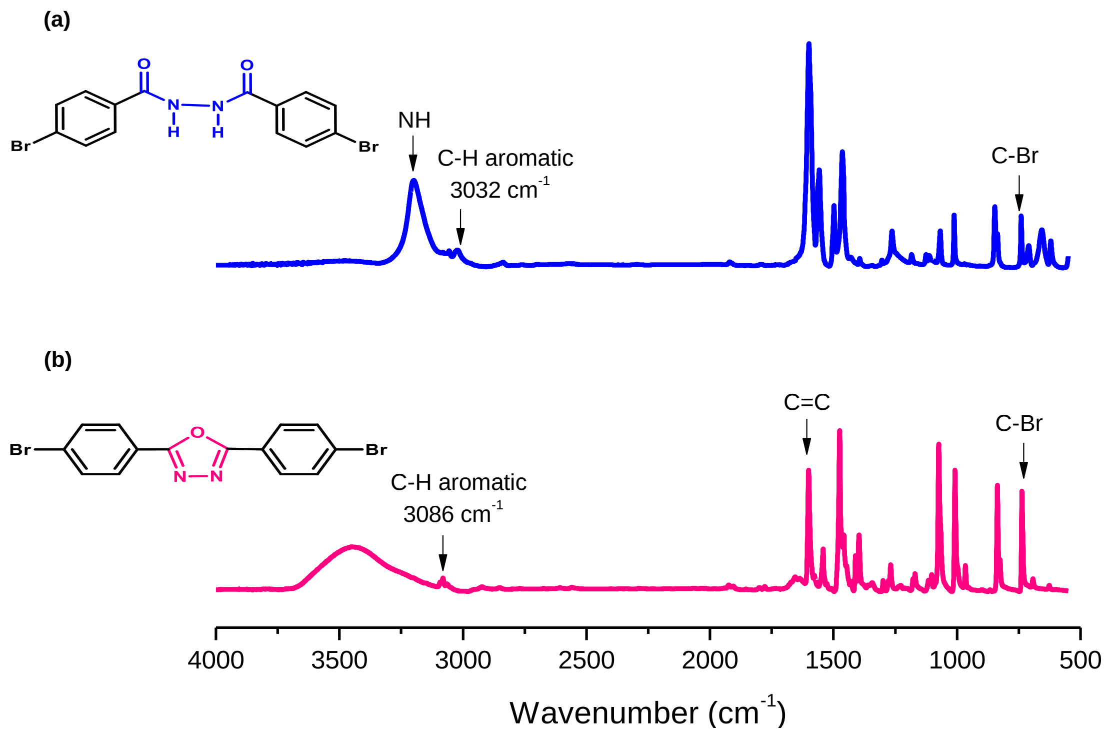

Figure S1. FTIR spectra of (a) *N,N′*-bis(4-bromobenzoyl)hydrazine and (b) OXD-Br2.

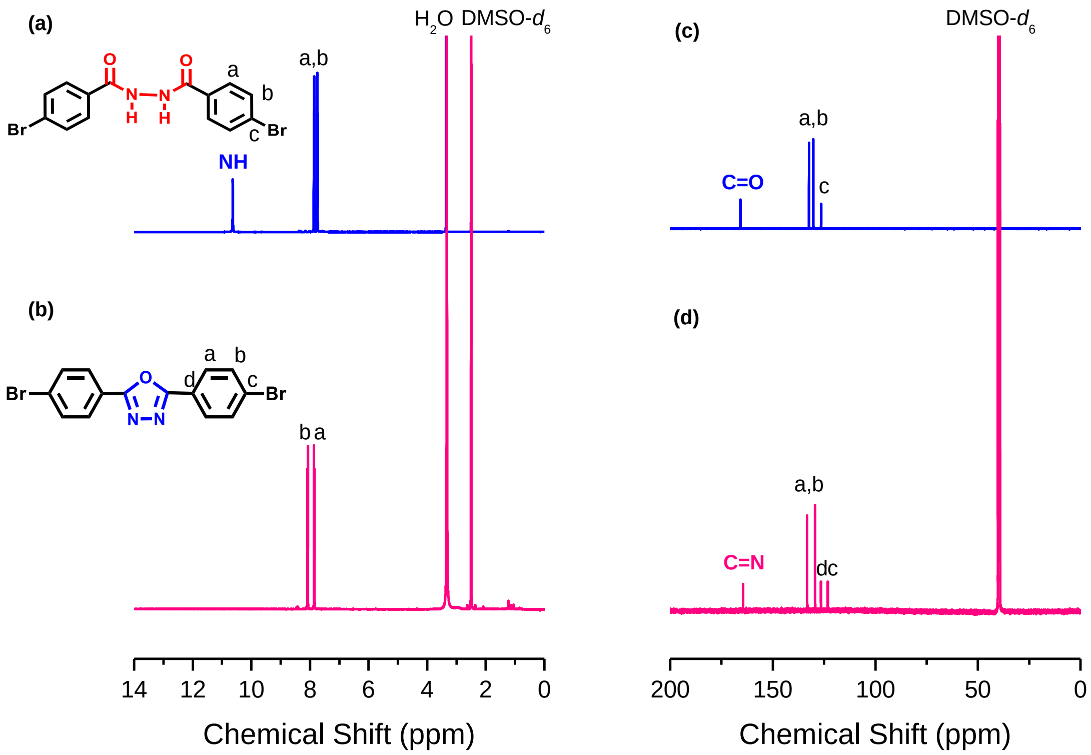

Figure S2. (a, b) ^{1}H and (c, d) ^{13}C NMR spectra of (a, c) *N,N′*-bis(4-bromobenzoyl)hydrazine and (b, c) OXD-Br2.

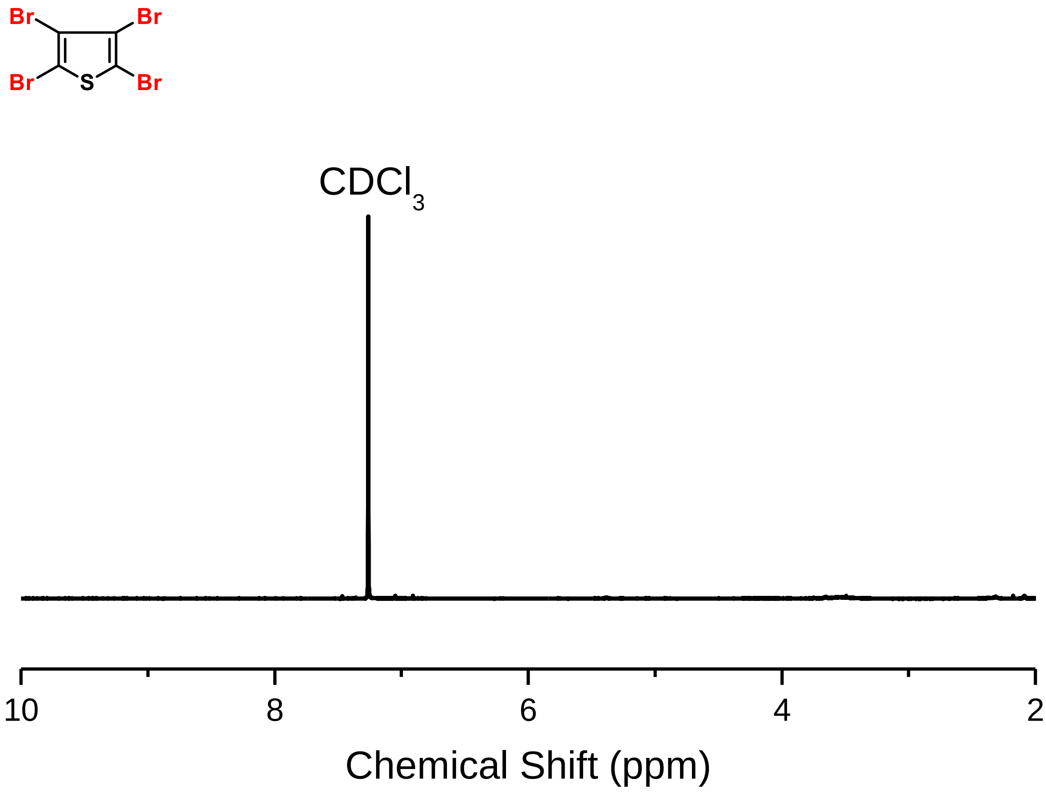

Figure S3. ^1H NMR spectrum of Th-Br_4.

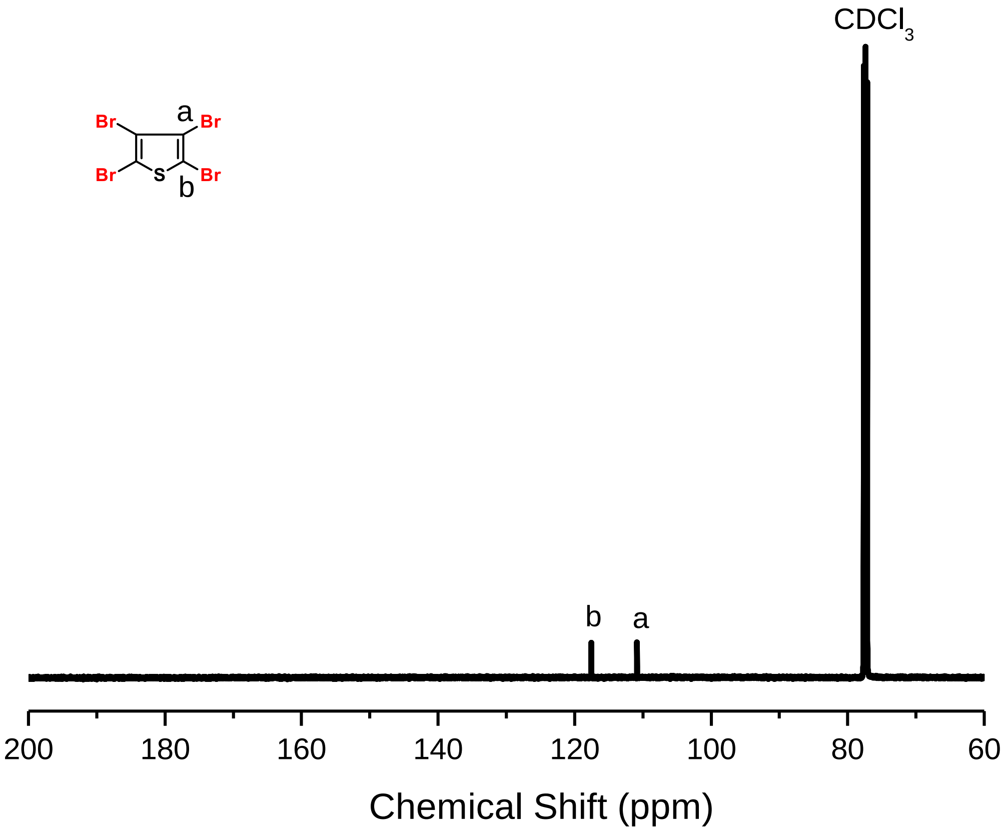

Figure S4. ^{13}C NMR spectrum of Th-Br_4.

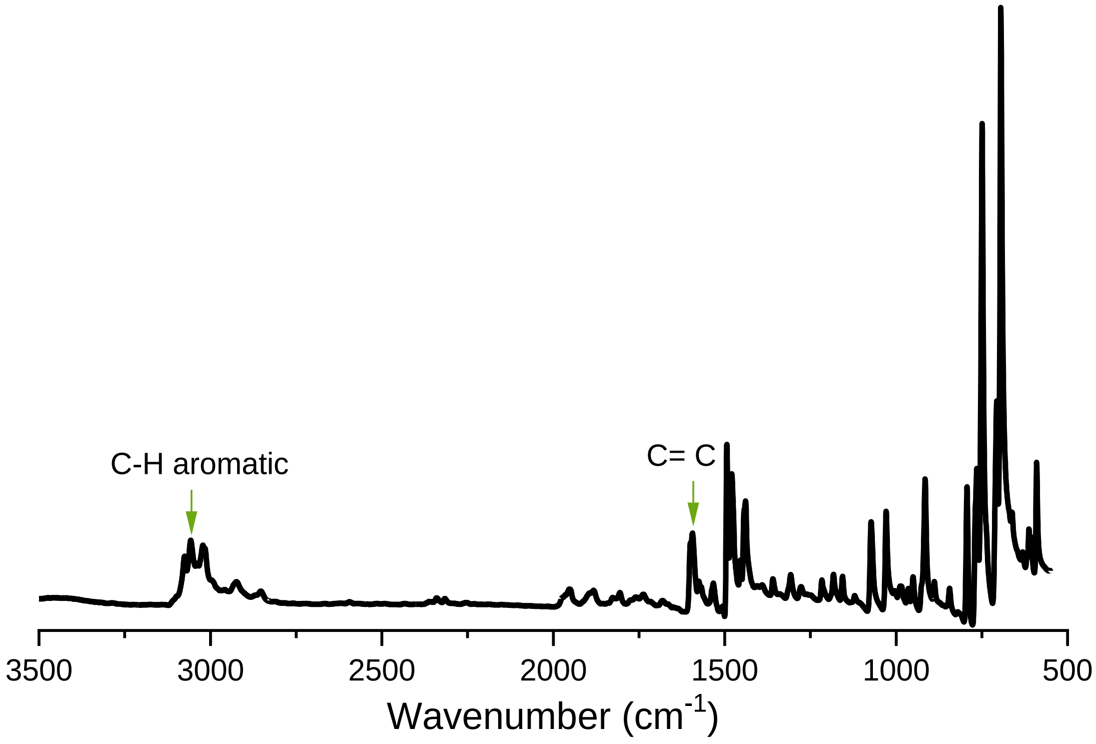

Figure S5. FTIR spectrum of TPTh.

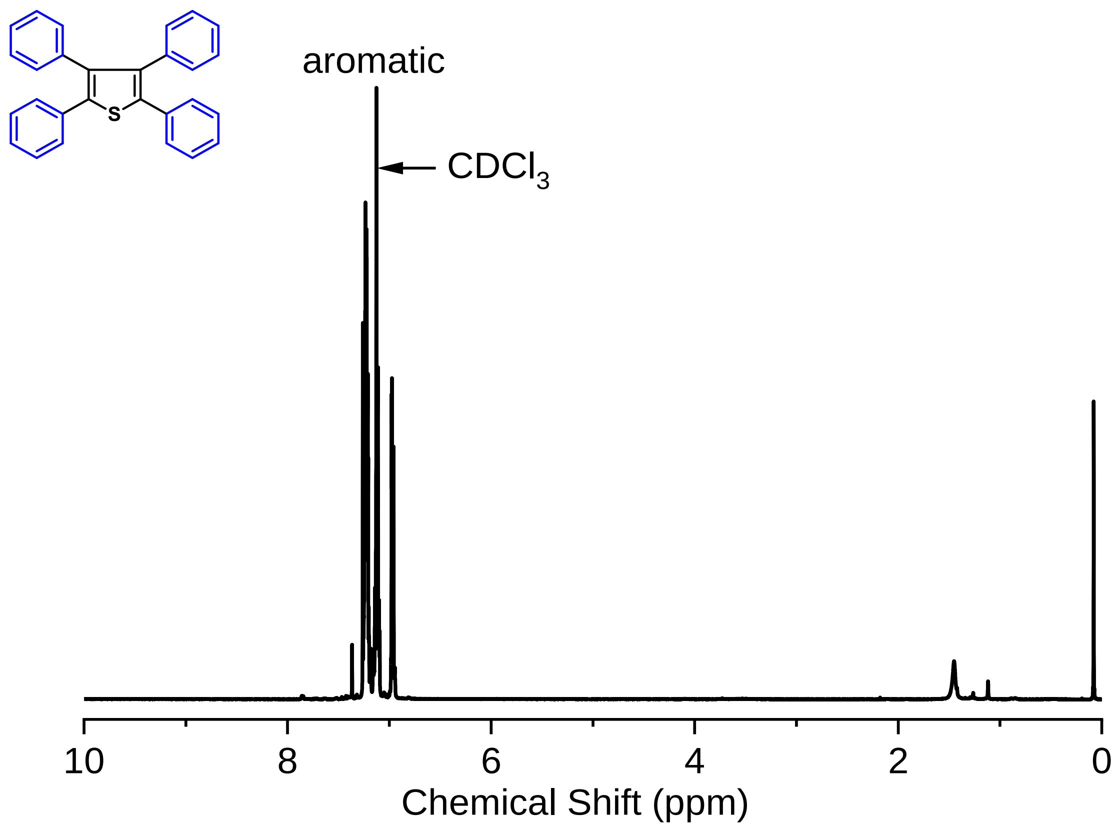

Figure S6. ^{1}H NMR spectrum of TPTh.

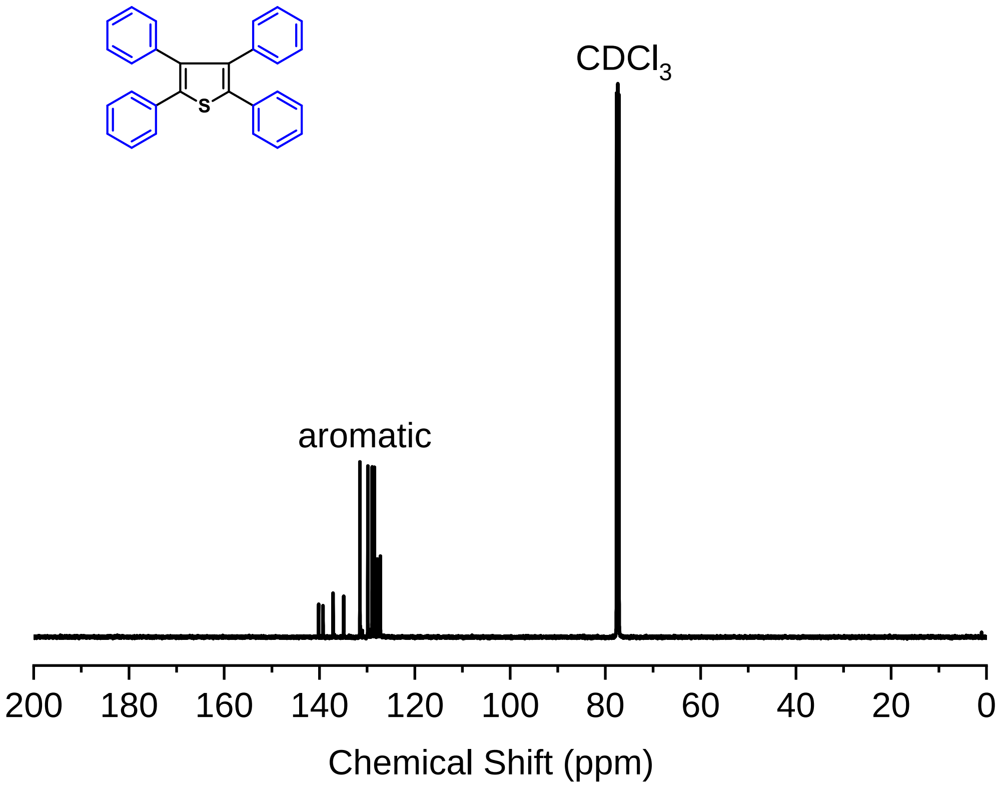

Figure S7. ^{13}C NMR spectrum of TPTh.

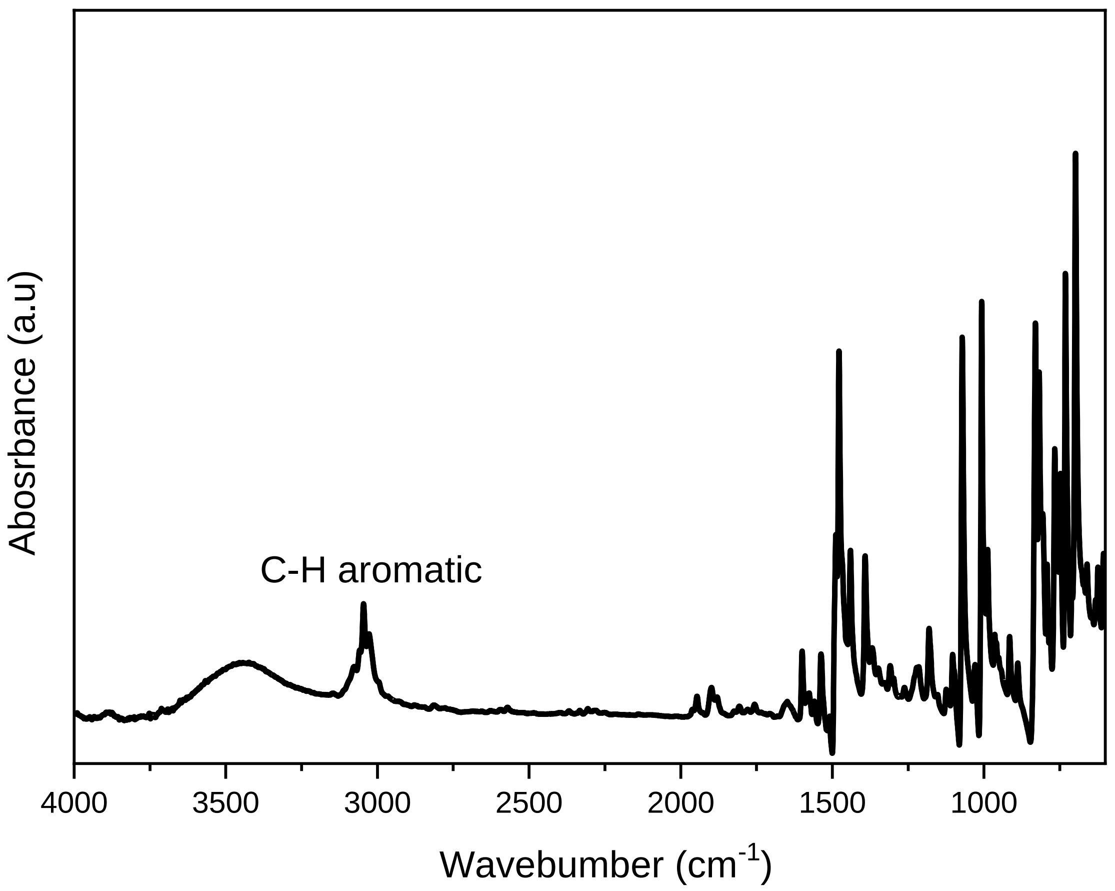

Figure S8. FTIR analysis of TPTh-Br_2.

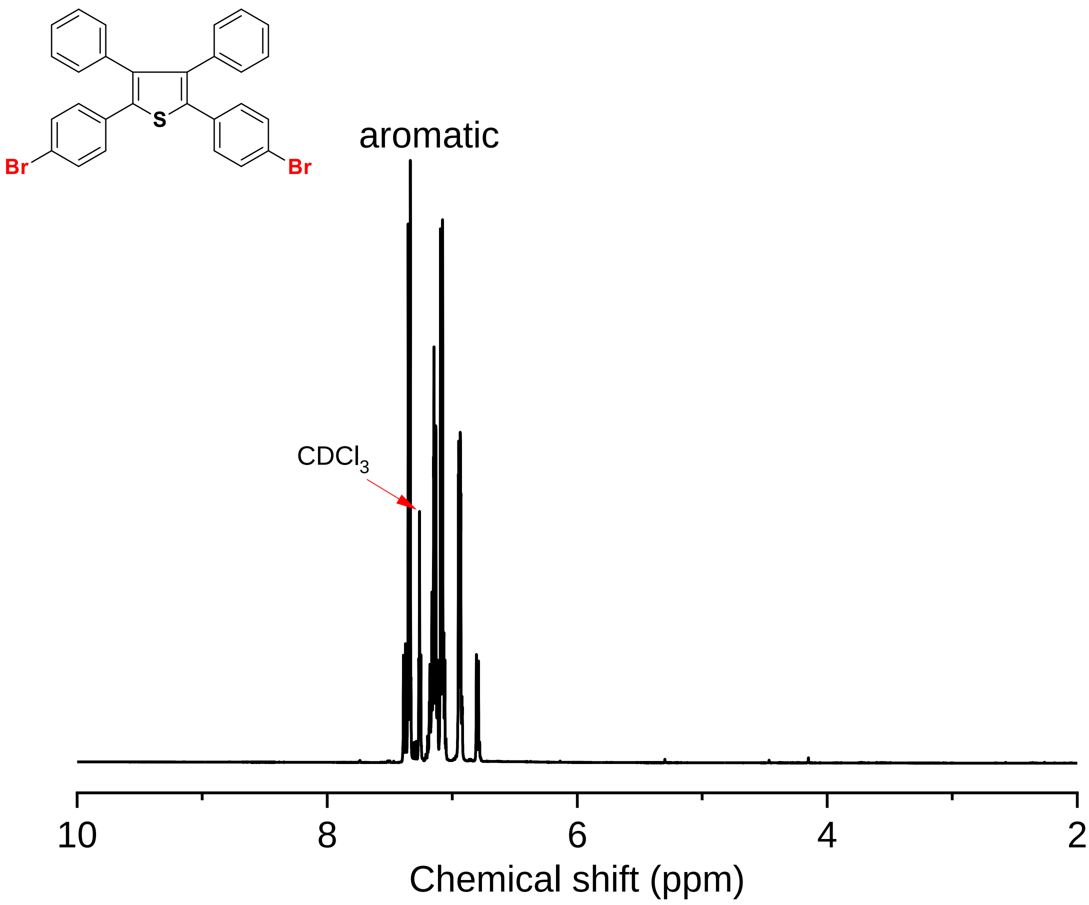

Figure S9. ^{1}H NMR analysis of TPTh-Br_2.

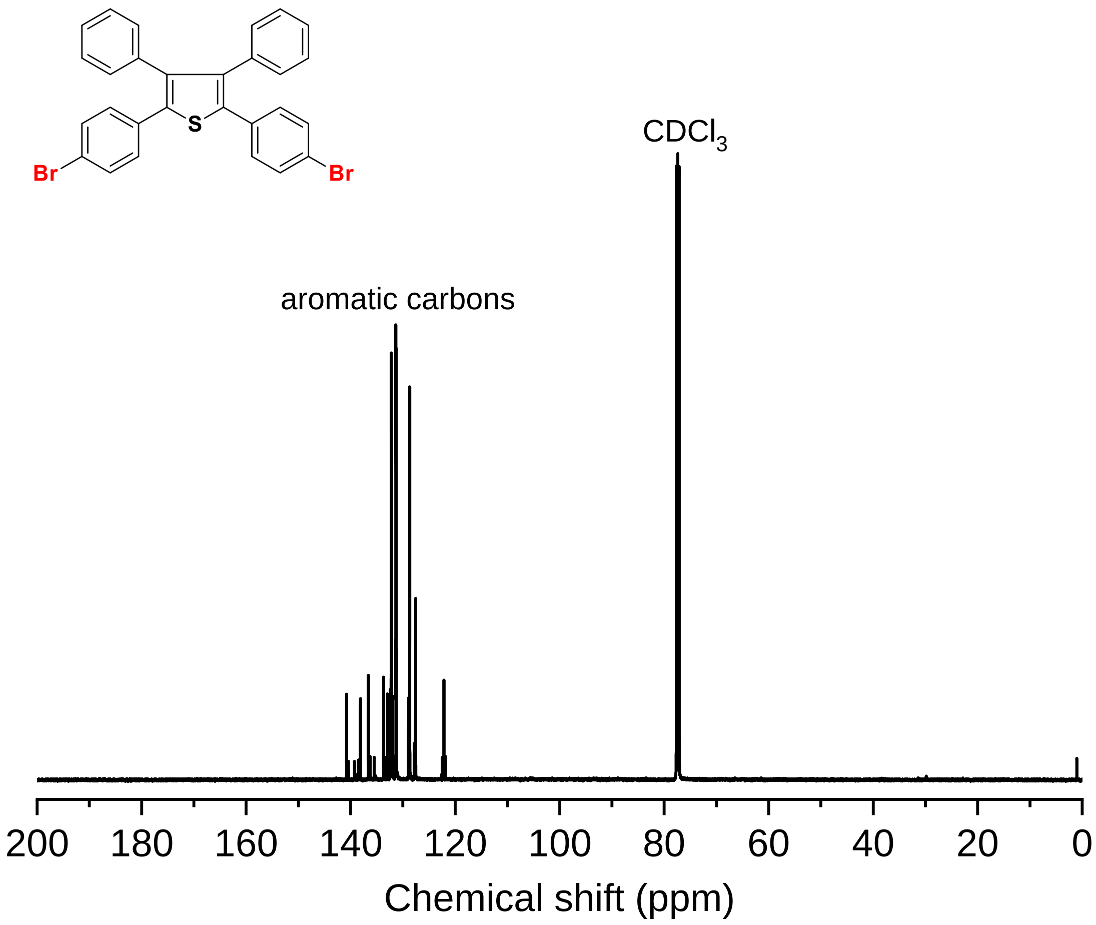

Figure S10. ^{13}C NMR analysis of TPTh-Br_2.

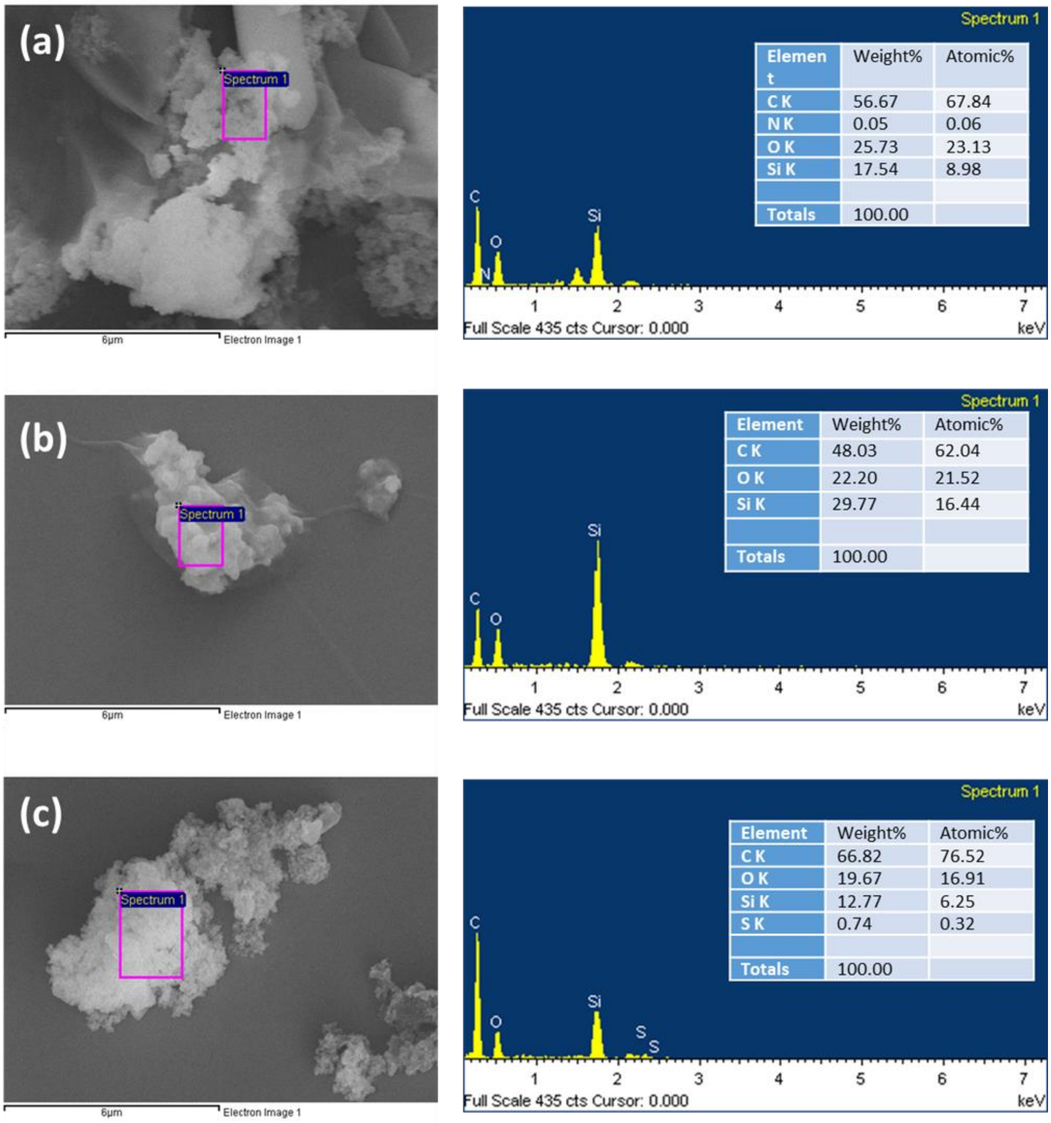

Figure S11. SEM and Elemental mapping (EDX) profile of the as-prepared (a) POSS-OXD POIP, (b) POSS-Th POIP and (c) POSS-TPTh POIP.

.

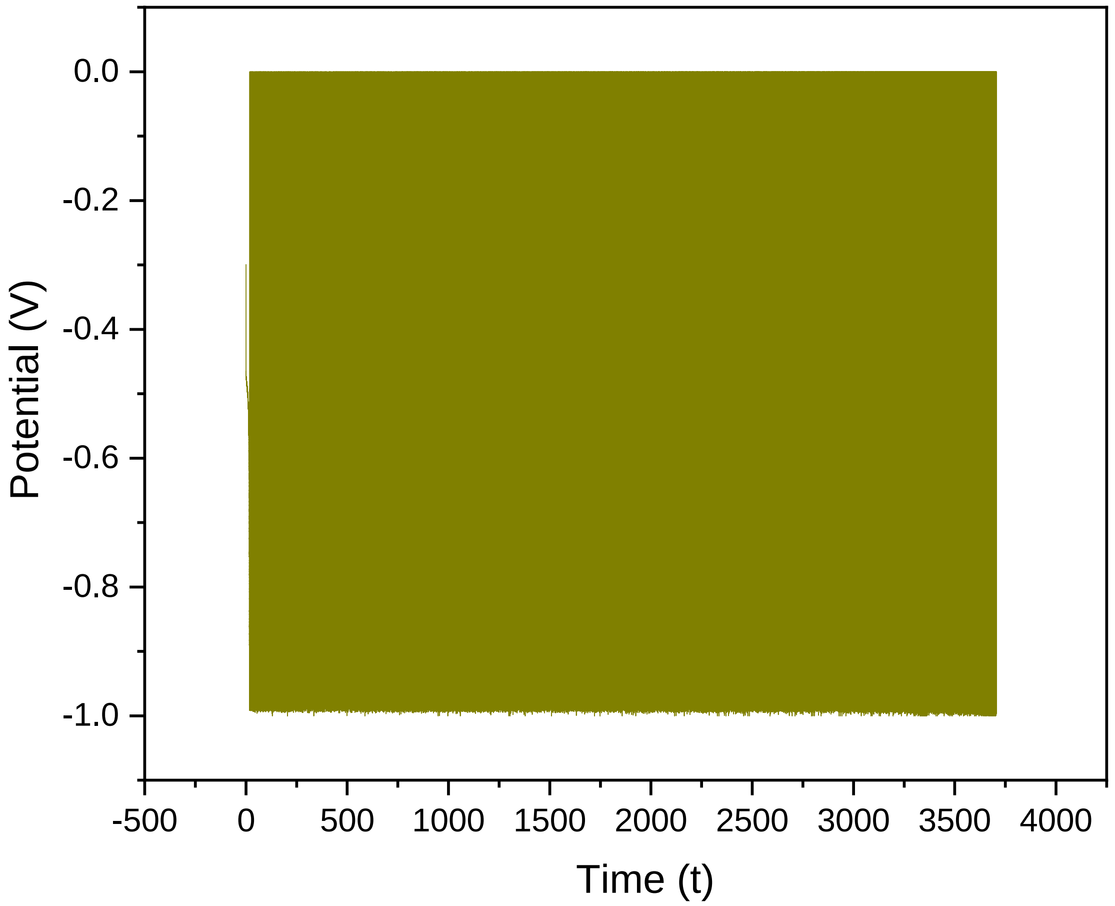

Figure S12. Stability test of the as-prepared POSS-OXD POIP.

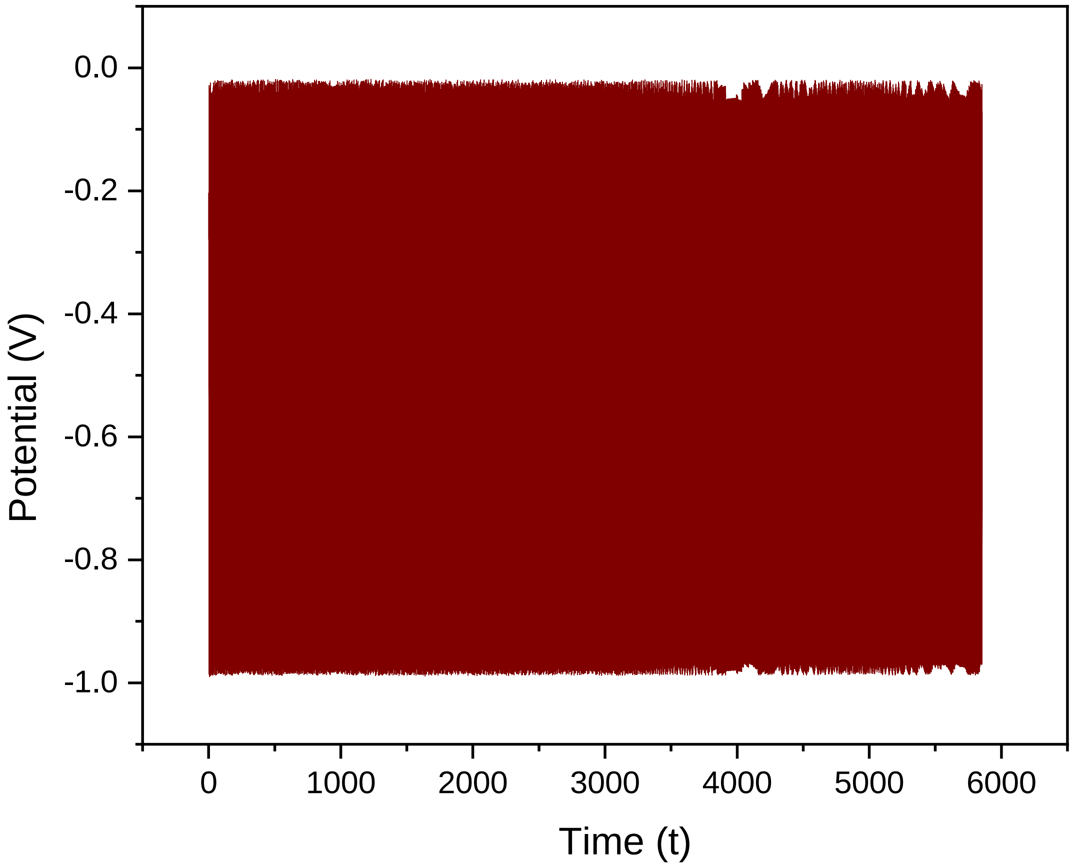

Figure S13. Stability test of the as-prepared POSS-Th POIP.

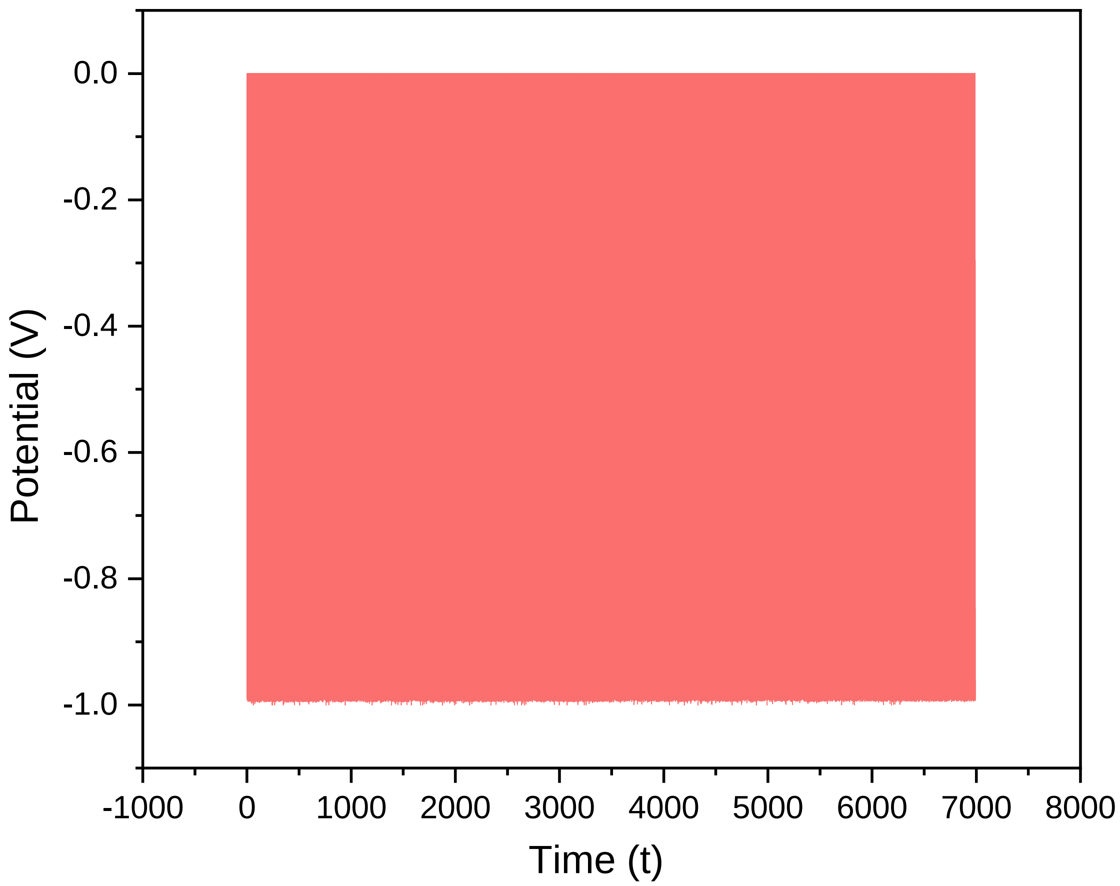

Figure S14. Stability test of the as-prepared POSS-TPTh POIP.

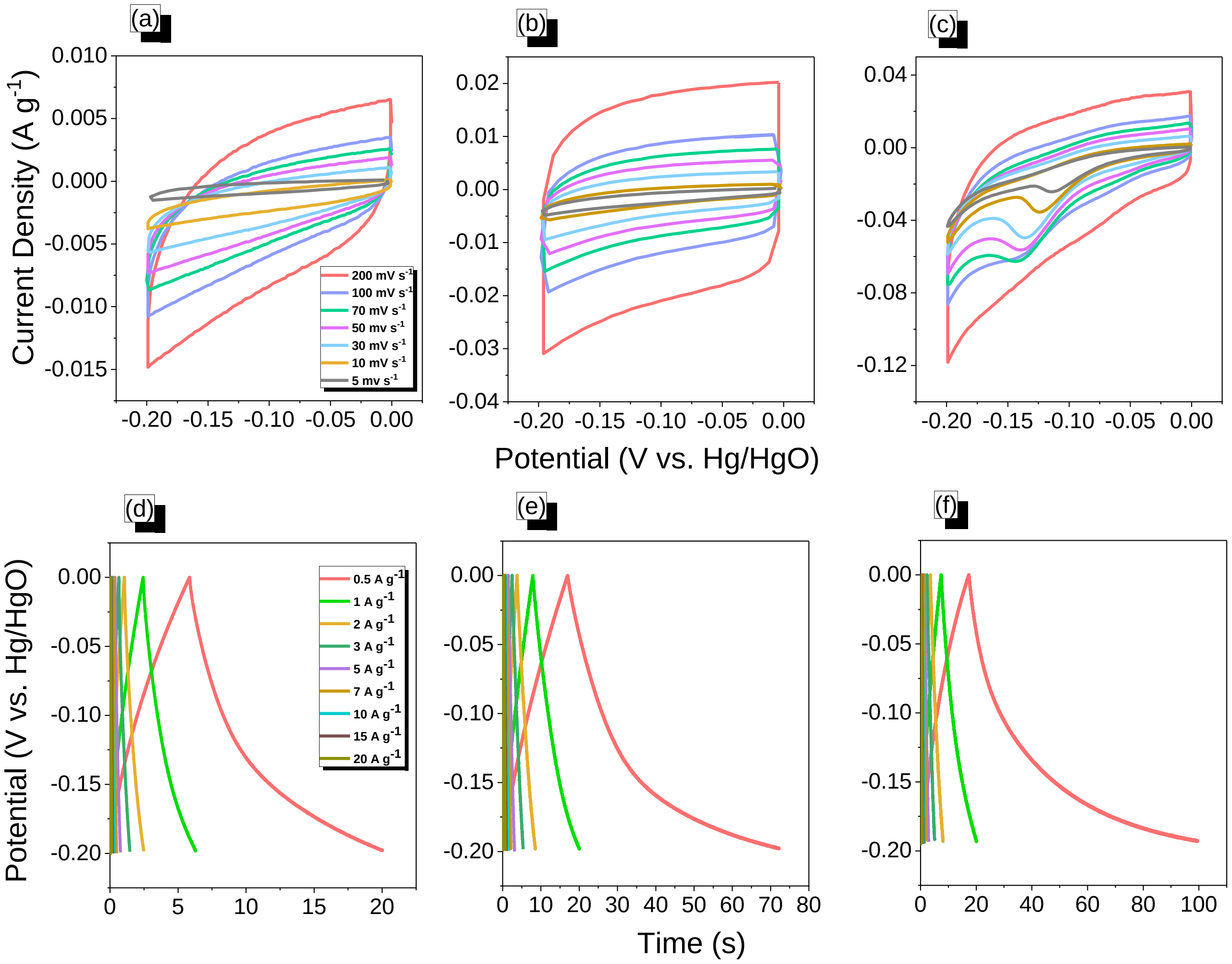

Figure S15. Cyclic voltammetry curves (a-c) and (d-f) galvanostatic charge-discharge profiles of POSS-OXD, POSS-Th and POSS-TPTh POIPs.

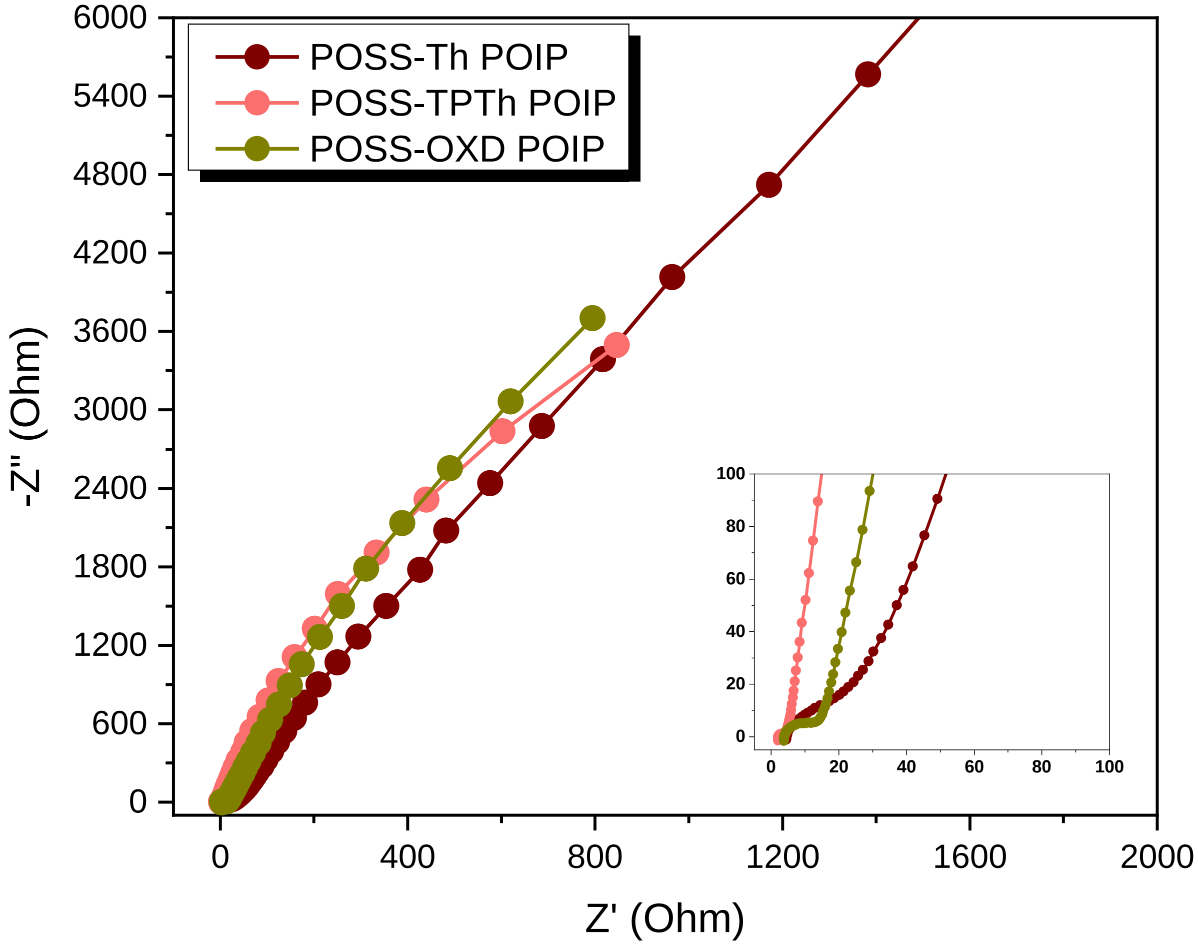

Figure S16. Nyquist plots of POSS-Th, POSS-TPTh, and POSS-OXD POIPs.
